# Poly-Lysine Dendritic Nanocarrier to Target Epidermal Growth Factor Receptor Overexpressed Breast Cancer for Methotrexate Delivery

**DOI:** 10.3390/ma15030800

**Published:** 2022-01-21

**Authors:** Pratibha Narayanan, Anju Krishnan Anitha, Neethu Ajayakumar, Kesavakurup Santhosh Kumar

**Affiliations:** Pathogen Biology Division, Rajiv Gandhi Centre for Biotechnology, Bio-Innovation Centre, KINFRA Park, Chanthavila (PO), Thiruvananthapuram 695585, Kerala, India; pratibhan@rgcb.res.in (P.N.); anjuka@rgcb.res.in (A.K.A.); neethua@rgcb.res.in (N.A.)

**Keywords:** poly-lysine dendritic nanocarrier, targeted drug delivery, breast cancer, methotrexate, epidermal growth factor receptor, in silico approach, solid-phase peptide synthesis, tetrapeptide spacer, in vivo evaluation

## Abstract

A fourth generation poly-lysine dendritic nanocarrier (P_4_LDN)-based targeted chemotherapy for breast cancer is attempted by incorporating an epidermal growth factor receptor (EGFR)-specific short peptide E2 (ARSHVGYTGAR) and the drug methotrexate (MTX) into a nanocarrier system. The drug is incorporated into the nanocarrier using a cathepsin B cleavable spacer: glycine–phenylalanine–leucine–glycine (GFLG). The in vitro analysis of the time-dependent drug release, binding and internalization ability, and the cytotoxic nature showed that this drug delivery system (DDS) is highly effective. The efficacy analysis using non-obese diabetic/severe combined immunodeficiency (NOD-SCID) mice also showed that compared to the control group, the DDS can effectively reduce tumor volume. The mice that received the DDS appeared to gain weight more rapidly than the free drug, which suggests that the dendrimer is more easily tolerated by mice than the free drug.

## 1. Introduction

The International Agency for Research on Cancer (IARC), in December 2020, reported that breast cancer is the most commonly occurring cancer in the world. According to the GLOBOCAN report, there were over 2.3 million cases and 685,000 deaths in the world in 2020 due to the fact of breast cancer. Chemotherapy is a better option for treating advanced breast cancers. But the narrow therapeutic index of the anticancer drugs and several off-target side effects associated with their biodistribution require further refinement to make it the best option for breast cancer treatment. Nanocarrier-based targeted drug delivery systems (DDS) provide enhanced selectivity, reduced drug-associated toxicity, improved drug solubility, and prolonged circulation in plasma [1]. Dendritic nanocarriers or dendrimers have gained wide attention over the past several years due to the fact of their high drug-loading capacity, low polydispersity, and high resistance to proteolytic degradation. Among the dendrimers that are utilized as drug delivery agents, lysine dendrimers may have a higher potency as drug carriers due to the fact of their biocompatibility, high water solubility, low polydispersity, and availability of various sidechain protections that can be removed under a set of chemistry that enables sufficient functional sites to incorporate the targeting moiety and the drug. In addition, as poly-lysine dendritic structures are made of lysine units, they are highly biodegradable and exhibit reduced cytotoxicity [2], and they have an ability to mimic cell-penetrating peptides for enhanced cellular uptake [3]. All these features indicate the high potential of poly-lysine dendritic structures for clinical application as a DDS.

Drug targeting can be achieved by two main techniques: passive as well as active drug targeting. Passive targeting is the passive accumulation of the drug or drug delivery agent, which is possible because of the leaky vasculature of the tumor microenvironment. Active targeting deploys several molecules that can target particular receptors or molecules at the cancer cell surface, thereby resulting in the selective accumulation of the drug. The overexpression of various receptors on cancer cell membrane is a characteristic feature that distinguishes cancer cells from physiologically normal cells. Epidermal growth factor receptor (EGFR) is one among the overexpressed receptors and, hence, they can be used as an effective target for delivering drugs. EGFR is a member of the ErbB family of receptor tyrosine kinases that play an important role in the pathogenesis of human cancers [4]. Herbst and co-workers, in 2006, evaluated the percentage of increased EGFR expression within some tumor types [5]. It was also reported that half of the cases of triple-negative breast cancer (TNBC) and inflammatory breast cancer (IBC) overexpress EGFR, and it is an attractive target for antitumor therapy [6]. There are several mechanisms by which cancer cells can obtain increased EGFR expression [7,8,9,10]. EGFR is regarded as a prototype of the EGFR family of receptors, which is endocytosed after ligand binding and activation and then transported to lysosomes for degradation [11]. Several anti-EGFR IgG monoclonal antibodies are commercially available, but they scarcely penetrate into solid tumors and have high manufacturing costs. Short peptides, though they have less therapeutic action than monoclonal antibodies, have several advantages such as being easily synthesized and modified, more economical, less toxic, highly biocompatible, and rarely immunogenic [12]. They can easily penetrate into the solid tumor area because of their small size. Identification of short peptides that specifically target the receptors overexpressed in solid tumors can be conducted by screening phage display libraries or by in silico methods [13,14]. 

Methotrexate (MTX) is an FDA approved drug used in the treatment of many cancers, including breast cancer, but the adverse effects related to its folate antagonistic action remain as a constraint to use this drug clinically. The dose-limiting toxicity of this drug includes hepatotoxicity and nephrotoxicity, but mortality is due to the fact of pneumonitis or secondary infections [15]. A potential approach to strengthen the efficacy of the administered drug without causing any side effects is to develop capable targeted DDS. In this study, we proposed to design and synthesize a fourth generation lysine dendritic nanocarrier based on the concept put forward by Helmut Ringsdorf in 1975 to be utilized as a drug delivery agent to deliver MTX [16]. To provide better target specificity, we anticipated to design and develop novel short peptides that can be incorporated into the selected peripheral functional sites of the nanocarrier as targeting moieties to target the EGFR. Five short peptides were designed in silico by replacing some amino acids that contribute less in the binding with other amino acids to fit a pre-defined structural template of known three-dimensional structures [17]. These short peptides were chemically synthesized by the SPPS technique using 9-fluorenylmethoxycarbonyl (Fmoc)-N^α^ protected amino acids [18]. Out of the five designed peptides, ARSHVGYTGAR (E2) was selected as the best peptide to target the EGFR overexpressed breast cancers. It was then covalently incorporated to half of the terminal amino groups of the lysine. The drug MTX was incorporated into the remaining half of the N-terminal amino groups, and all sidechain amino groups through a tetrapeptide spacer, GFLG (G). Mehta and co-workers demonstrated that when poly-lysine dendrimers were used to deliver doxorubicin through a self-emolative diglycolic acid-V-Citrulline linker in lung and breast cancers, more control over in vivo drug release profiles was seen, and it also resulted in limited systemic side effects [19]. In our study, the drug release experiment proved that the enzyme cathepsin B selectively cleaved this spacer and resulted in a slow and gradual release of MTX from the carrier. We also confirmed the targeted action of the DDS towards EGFR through flow cytometry experiments and confocal imaging. The anti-tumor efficacy analysis in NOD-SCID mice revealed that there was a reduction in tumor volume compared to the control in the case of a DDS than free drug. All these results highlight the superior efficacy of our DDS over the free drug.

## 2. Materials and Methods

### 2.1. Materials

#### 2.1.1. Chemicals

Fmoc amino acids and cross-linked ethoxylate acrylate resin (CLEAR) were purchased from NovaBiochem^®^, Merck Life Science Private Limited (Mumbai, India). Hydroxy methyl-3-methoxy phenoxy-acetic acid (rink amide) linker was obtained from Fluka (Buchs, Switzerland). 1-Hydroxybenzotriazole (HOBt) and 2-(1H-benzotriazol-1-yl)-1,1,3,3, tetramethyluronium hexafluorophosphate (HBTU) were procured from Peptides International (Louisville, KY, USA). Dimethyl formamide (DMF), ethanedithiol, piperidine, ninhydrin, diethyl ether (DEE), and diisopropylethylamine (DIEA) were from Spectrochem Private Limited (Mumbai, India). Triisopropyl silane, methotrexate hydrate, and cathepsin B (from human placenta) were purchased from Sigma–Aldrich (St. Louis, MI, USA). Fluorescein methotrexate and Hoechst 33342 dye were obtained from Invitrogen^TM^, Thermo Fisher (Waltham, MA, USA). 

#### 2.1.2. Cells, Media, Supplements, Stains, and Antibodies

The cell lines MDA-MB-231, SiHa, and HEK-293T were obtained from NCCS, Pune, India. They were cultured in Dulbecco’s modified Eagle’s medium (DMEM; Invitrogen, Waltham, MA, USA), supplemented with 10% fetal bovine serum (FBS; Gibco^TM^, Thermofischer, Waltham, MA, USA), penicillin (50 units/mL), and streptomycin (50 µg/mL) (Penstrep; Thermofischer, Waltham, MA, USA) at 37 °C under a humidified atmosphere containing 5% CO_2_. 4, 6-Diamidino-2-phenylindole (DAPI) and MTT (3-(4,5-dimethylthiazol-2-yl)-2,5-diphenyl tetrazolium bromide) dye were procured from Sigma–Aldrich (Milwaukee, MO, USA). Human EGFR antibody and donkey anti-goat IgG (PE-conjugated antibody) were purchased from R&D systems (Minneapolis, MI, USA). Propidium iodide and RNase were procured from Abcam (Cambridge, UK).

#### 2.1.3. Animals

The animal experiment protocols were approved by the Institutional Animal Ethics Committee (IAEC/773/KSAN/2019). Randomized female mice of NOD-SCID strain (weighing approximately 15–25 g) were selected and housed in an individually ventilated caging system and maintained at an ambient temperature of 23 °C and a humidity of 50–70%. They were fed with irradiated commercial pelleted mice feed ad libitum and autoclaved water ad libitum. The guidelines for performing animal experiments were approved by the Animal Ethics Committee, Rajiv Gandhi Centre for Biotechnology, Thiruvananthapuram. Animals were observed daily for mortality or any unhealthy symptoms prior to the commencement of the study. When they attained 3–4 weeks of age, they were used for the experimental purpose. All the techniques used for mouse handling, breeding, injection, and sacrifice were performed in strict accordance with the guidelines of the Government of India’s Committee for the Purpose of Control and Supervision of Experiments on Animals (CPCSEA).

### 2.2. Synthesis of Poly-Lysine Dendritic Nanocarriers of the Fourth Generation (P_4_LDN)

P_4_LDN was synthesized on CLEAR base resin (0.37 mmol/g capacity) using an orthogonal protection strategy. The two different lysines used were fmoc-lys(mtt)-OH and fmoc-lys(fmoc)-OH. Twenty percent piperidine for 30 min was used to remove the fmoc group, and 5% TFA in DCM for 30 min was used to remove the mtt group. The coupling and deprotection reactions were monitored by ninhydrin test. After the overnight swelling of the resin in DMF, it was treated with DIEA, and the rink amide linker was attached to it covalently with the help of coupling reagents HOBt, HBTU, and DIEA. First coupling was given using a linker (3.5 millimoles) and coupling reagents (3.5 millimoles) for 1 h, and the second coupling was given by adding a linker and coupling reagents (1.75 millimoles with respect to the resin capacity) for 30 min. After the attachment of the rink amide linker to the resin, the fmoc group of the linker was removed using piperidine and the first amino acid, fmoc-lys(mtt)-OH, was covalently attached with the help of the coupling reagents by the fmoc strategy two times successively. The fmoc group of the second lysine was then removed using 20% piperidine. The mtt groups of both the first and second amino acids were retained in this step. Then, the third amino acid, fmoc-lys(fmoc)-OH, was covalently attached; both fmoc groups were removed by 20% piperidine. This created a branch point, and to the two terminal amines of the branch, fmoc-lys(mtt)-OH (3.5 millimoles for first coupling and 1.75 millimoles for second coupling) was attached two times successively. The steps for the coupling and deprotection were repeated until the fourth generation was obtained. For the third and fourth generations, amino acids and coupling reagents (5 millimoles, 8 couplings of 1 h each) were given for successful coupling. After the synthesis of the fourth generation dendrimer, a small portion of the P_4_LDN was cleaved from the resin to confirm the molecular mass. For that, fmoc deprotection of the terminal amino group and the mtt deprotection of the sidechain amino groups were conducted using piperidine and 5% TFA-DCM, respectively. Then the resin was thoroughly washed with DCM, dried using DEE and kept under vacuum desiccator overnight. Cleavage was carried out using TFA/TIS/water (95:2.5:2.5) for 3 h, and the peptide dendritic molecule was collected in a round-bottomed flask. The solvent was then evaporated using a rotary vacuum evaporator, and the peptide dendritic molecule was precipitated using ice-cold DEE. It was washed thoroughly in DEE by centrifugation to collect the pellet and dissolved in buffer A containing 0.1% TFA in water.

### 2.3. Characterization of P_4_LDN

P_4_LDN was purified by C18 reversed-phase high-performance liquid chromatography (RP-HPLC). The programme was run by increasing the buffer B (0.08% TFA in acetonitrile/water (80:20)) concentration to 100% in 20 min with a flow rate of 1 mL/min. Peptide (10–20 pmole) was dissolved in a saturated solution of α-Cyano-4-hydroxycinnamic acid, loaded to sample plate, dried, and analyzed by MALDI for determining the mass. The peptide fraction with a similar mass (confirmed by MALDI-TOF MS) was collected by C18 RP-HPLC. The FTIR (KBr-IR) spectrum was recorded using a PerkinElmer Spectrum Two instrument in a wavenumber range between 4000 and 500 cm^−1^ by running 5 scans with a resolution of 4 cm^−1^. For ^1^H NMR spectroscopy, the sample was dissolved in DMSO, and ^1^H NMR spectra were obtained by the measurement of radiation intensity as a function of wavelength using a Bruker Avance III 400 MHz NMR instrument. The size and morphology of P_4_LDN was observed by transmission electron microscopy (TEM). The sample was dispersed in ethanol and loaded on formvar-coated copper grids (200 mesh size, Electron Microscopy Sciences (Hatfield, PA, USA)). The photomicrographs were taken at suitable magnifications.

### 2.4. Selection of the Best EGFR-Targeting Short Peptide Ligands In Silico and In Vitro

The structure of the receptor in complex with the ligand was extracted from Protein Data Bank. The ligand binding region in the receptor and the amino acid residues in the ligand that take part in the binding were determined using PyMOL. The average contribution of these EGF residues to the binding energy was then calculated by GROMACS molecular dynamics (MD) simulation studies. Peptides were designed by replacing the amino acids that showed the least contribution to the binding energy by other amino acids having similar characteristics. Preliminary docking studies were conducted using PatchDock software, where both the receptor and the peptide were considered as rigid. The peptides with good binding scores (lowest binding energy) were further subjected to Rosetta FlexPep ab initio docking (RMSD) to perform local refinement of the best estimated conformation of a peptide in complex with receptor using a flexible sidechain, and the free energy scores were identified. The peptides with a good binding score were selected for further simulation studies. GROMACS 5.1.4 software was used for the MD simulation of the peptides with EGFR with a time scale of 20 ns. The total binding free energies (kJ/mol) of the EGFR–peptide complexes were calculated using molecular mechanics-Poisson–Boltzmann surface area (MM-PBSA) method.

### 2.5. Attachment of Peptide E2, GFLG Spacer, and MTX Drug to P_4_LDN

After the synthesis of P_4_LDN, the fmoc protection group was removed from the N-terminal, and the amino acids of the peptide E2 was covalently attached one by one to the N-terminal by the fmoc strategy using 3.5 equivalents of coupling reagents HBTU, HOBt, and DIEA. Half of the required number of amino acids were added so that half of the N-terminal amino groups of P_4_LDN remained free, where the drug molecule would be attached later. Then, the mtt groups in the sidechain amino groups of P_4_LDN were removed using 5% TFA in DCM. To the remaining N-terminal amino groups and sidechain amino groups, the spacer GFLG was attached covalently by fmoc strategy, followed by the covalent attachment of the drug methotrexate (5 equivalents) using the solid-phase synthesis strategy. The carboxyl group of MTX was activated using the coupling reagents HBTU, HOBt, and DIEA and attached to the free amino group of the amino acid G of the cleavable spacer GFLG. The coupling and deprotection reactions were confirmed by ninhydrin test. Finally, MTX–G–P_4_LDN–E2 was cleaved from the resin, precipitated, washed, and lyophilized.

### 2.6. Characterization of MTX–G–P_4_LDN–E2

MTX–G–P_4_LDN–E2 was purified by C18 RP-HPLC, and the mass was analyzed using MALDI-TOF MS. The FTIR spectra were collected in the 4000–500 cm^−1^ wavenumber ranges by running 5 scans with a resolution of 4 cm^−1^. The samples were prepared in phosphate-buffered saline (PBS, pH 7.4), and the hydrodynamic sizes, polydispersity indexes (P.D.I) and zeta potentials of MTX–GP4D–E2 and MTX–GP4D–V were measured using a Beckman Coulter Delsa^TM^ Nano C particle analyzer. The morphology of MTX–G–P_4_LDN–E2 was analyzed by TEM.

### 2.7. Determination of Drug Concentration in the Nanocarrier System

The concentration of MTX in the MTX–G–P_4_LDN–E2 system was calculated using C18 RP-HPLC at an excitation wavelength of 370 nm and an emission wavelength of 470 nm. Dilutions of MTX stock solution (1 mg/mL) was made in PBS (pH 7.4) to prepare different concentrations of MTX. The standard graph was plotted with concentration on the *x*-axis and the peak area on the *y*-axis. Then, the concentration of MTX in MTX–G–P_4_LDN–E2 was calculated by plotting the area on the standard graph.

### 2.8. Estimation of Enzymatic Drug Release from MTX–G–P_4_LDN–E2 

The release of MTX from MTX–G–P_4_LDN–E2 in the presence and absence of the CTSB enzyme was estimated using the C18 RP-HPLC method. The system (4 mg/mL) was incubated with 5.6 µM CTSB enzyme (in 0.1 M phosphate buffer, pH 6 with 0.01 M EDTA and 0.05 M reduced glutathione). The system without the enzyme was taken as the control. The solutions were continuously stirred and maintained at 37 °C. At different time intervals (0.5, 1, 2, 3, 4, 7, 24, and 30 h), small volumes of sample were taken and centrifuged for 5 min at 10,000 rpm to collect the supernatant. The supernatant was immediately injected into the C18 RP-HPLC column (MTX: excitation 370 nm, emission 470 nm) and the cumulative percentage of MTX released from the system was plotted against the incubation time [20].

### 2.9. Evaluation of the Receptor Binding Abilities of MTX–G–P_4_LDN–E2

MDA-MB-231 (EGFR overexpressed) and SiHa (EGFR negative) cells were seeded into 6-well plates at a density of 1 × 10^5^ cells/well. Then fluorescein MTX (5 µg/mL) and fluorescein MTX-conjugated systems (8.6 µg/mL; having equivalent MTX concentrations) were added to each well and incubated for 24 h. After incubation, the cells were trypsinized and analyzed by flow cytometry. The cells without MTX or DDS treatment were taken as the control.

### 2.10. Functional Evaluation of MTX–G–P_4_LDN–E2 

MDA-MB-231 (EGFR overexpressed) and SiHa (EGFR negative) cells were grown on coverslips inside the 6-well plates, and the receptor binding ability of MTX–G–P_4_LDN–E2 was analyzed by confocal microscopy. Fluorescein methotrexate (5 µg/mL) and fluorescein MTX conjugated system (8.6 µg/mL; having equivalent methotrexate concentrations) were added to the wells and incubated for 24 h. The cells without MTX or DDS treatment were taken as the control. Then, Hoechst 33342 (10 µg/mL) was added and incubated for 15 min. The cells were observed under a confocal laser scanning microscope at suitable magnifications.

### 2.11. Internalization of MTX–G–P_4_LDN–E2

MDA-MB-231 (EGFR overexpressed) cells were seeded into a 6-well plate at a density of 1 × 10^5^ cells/well. The cells were serum starved for 5 h by incubation at 37 °C in DMEM only. The cells were trypsinized, washed with PBS, and then incubated with MTX–G–P_4_LDN–E2 (100 μg/mL; made in DMEM) and kept at 37 °C for 1 and 2 h for internalization. Serum starved cells treated with media lacking the DDS were used as the control. After the specified time, the cells were blocked with BSA for 20 min, followed by the addition of primary EGFR antibody and incubated at room temperature for 30 min. The cells were washed thrice with PBS, followed by the addition of the PE-conjugated secondary antibody. The cells were washed thrice, rapidly placed on ice to halt receptor trafficking, and then washed three times with ice-cold PBS for 5 min each on a shaking platform to remove excess nanocarrier system. Non-internalized systems were removed from the cell surface using three 5 min washes in ice-cold acid stripping buffer followed by three washes (5 min each) in ice-cold PBS [21]. The cells were then resuspended in media and analyzed by flow cytometry and BD FACS software for calculating the mean fluorescence intensity (MFI). At two time points (1 and 2 h of incubation), the MFI was compared with the untreated control cells and calculated using the following equation:

MFI (internalized nanocarrier drug delivery system) = MFI (Control)−MFI (at each time point).

### 2.12. Cytotoxicity Analysis by MTT Assay

The cytotoxic efficacy of MTX–G–P_4_LDN–E2 was analyzed by an MTT assay. The cells were seeded in a 96-well plate at a density of 5000 cells per well. The EGFR-targeting MTX–G–P_4_LDN–E2 was added in two different concentrations (4.3 and 8.6 µg/mL; equivalent to the free MTX) in MDA-MB-231 and SiHa cells. After 48 h of incubation, the cells were washed twice with 1X PBS, followed by the addition of MTT reagent (100 μL; 5 mg/mL in PBS) and 100 μL serum-free media to all wells and incubated for 3 h. Then DMSO (100 µL) was added to solubilize the formazan crystals, formed in viable cells. After 20 min, the optical density (OD) was measured at 570 nm using a microplate reader (BioRad). The percentage cell viability was calculated as 100 percentage cytotoxicity, where:

$$\%\;\mathrm{cytotoxicity}=\frac{O.D\;\mathrm{of}\;\mathrm{control}-O.D\;\mathrm{of}\;\mathrm{test}\;}{O.D\;\mathrm{of}\;\mathrm{control}}\times 10$$

### 2.13. Cell Cycle Analysis 

The cell cycle analysis was conducted by flow cytometry using the manufacturer’s protocol. Briefly, MDA-MB-231 and SiHa cells (1 × 10^5^cells/well) were incubated with MTX and MTX–G–P_4_LDN–E2 for 24 h, trypsinized, and centrifuged to collect the pellet. The cells were then fixed with pre-cooled 70% ethanol at 4 °C for 45 min. After fixation, the pellet was collected carefully after centrifugation at 850× *g* for 5 min. It was then washed twice with 1X PBS, and 50 μL RNase (100 μg/mL) was added and incubated for 45 min at 37 °C. Then, the cells were washed with 1X PBS and stained with propidium iodide (100 μL, 50 μg/mL) for 10 min. The cellular DNA was analyzed by flow cytometer, and the percentage of cells in each phase of the cell cycle was analyzed by BD FACS Diva software.

### 2.14. Orthotopic Model Development and Tumor Treatment Studies

For analyzing the efficacy of the nanocarrier systems, fifteen male and fifteen female NOD-SCID mice were assigned to three groups each (*n* = 3 in first group and *n* = 6 in second and third groups). Tumor was implanted by injecting cells subcutaneously in the dorsolateral flank with a concentration of 5 × 10^6^ MDA-MB-231 cells to female NOD-SCID mice. When the volume of tumor reached 500–1000 mm^3^, the first group of animals, serving as the control group, was given PBS (5 mL/kg body weight). The second group was given free-drug MTX (20 mg/kg) through the tail vein. The third group received MTX–G–P_4_LDN–E2 (35 mg/kg, equivalent to the MTX concentration) through the tail vein, and the treatment was given to the mice 5 times for 20 days.

### 2.15. Anti-Tumor Evaluation and Efficacy Studies of the Dendrimer Drug Delivery System

The tumor volume and body weight of the animals were measured before each injection. The body weight and tumor volume on the final day (24th day) was also calculated. A vernier caliper was used to measure the tumor, and the tumor volume was calculated using the formula:

$$\mathrm{Tumor}\;\mathrm{volume}\;\left({\mathrm{mm}}^{3}\right)=\frac{\left(\mathrm{length}\;\mathrm{in}\;\mathrm{mm}\right)\times {\left(\mathrm{width}\;\mathrm{in}\;\mathrm{mm}\right)}^{2}}{2}$$

### 2.16. Statistical Analysis

All the data are expressed as arithmetic mean ± standard deviation. Statistical analysis of data was conducted using one-way ANOVA followed by Dunnett’s post hoc test (for in vivo experiments) and Tukey’s test (for internalization assay) using GraphPad Prism 6. Statistical significance of the differences was considered at a *p*-value < 0.05 (for in vivo experiments and a *p*-value < 0.005 (for internalization assay).

## 3. Results

### 3.1. Characterization of P_4_LDN 

**Scheme 1:**
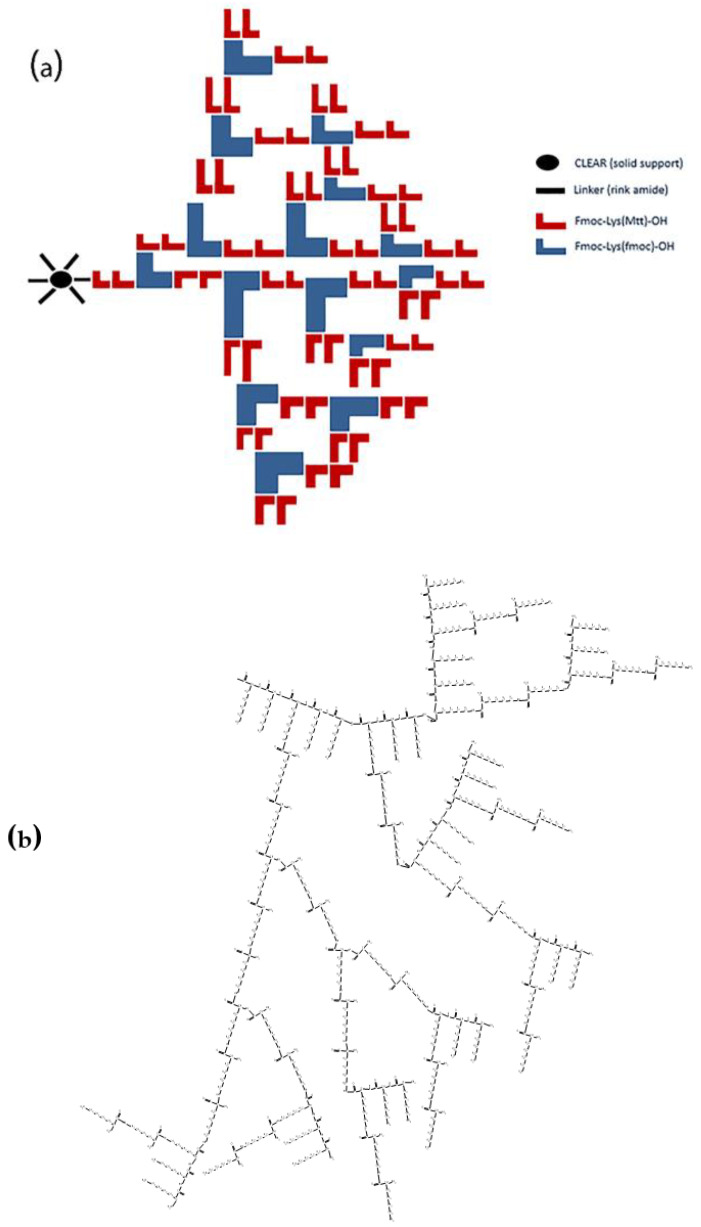
(**a**) Synthesis protocol (attachment of lysine units is shown only in one functional site of the linker); (**b**) chemical structure of P_4_LDN after cleavage from the resin.

The illustration of P_4_LDN was generated using ChemDraw Ultra 12.0 software (Scheme 1a,b). They were then synthesized by SPPS using an orthogonal protection strategy and purified by RP-HPLC.

**Figure 1:**
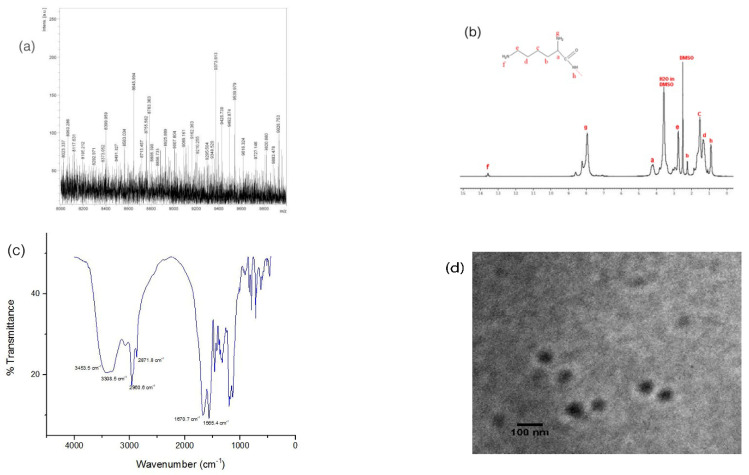
(**a**) MALDI-TOF MS profile: The [M+H]^+^ value calculated for C_456_H_1030_N_132_O_63_ was 9372.86 Da and found to be 9373.613 Da; (**b**) NMR spectra (400 MHz, DMSO-*d*_6_), ppm: 0.911 (s, 24H, NH), 1.343, 1.538, 2.253 (m, 154H, CH_2_), 2.762 (m, 51H, CH_2_–NH_2_), 4.204 (s, 21H, CH), 7.943, 8.232, 8.599 (t, 80H, NH2), and 13.584 (s, 2H, NH2); (**c**) FTIR spectra: wavenumber = 3453.5 cm^−1^ (m), 3308.5 cm^−1^ (m), 2960.6 cm^−1^ (m), 2871.8 cm^−1^ (m), 1670.7 cm^−1^ (s), and 1565.4 cm^−1^ (s); (**d**) TEM image of P_4_LDN.

MALDI-TOF MS analysis showed that molecular weight of P_4_LDN was 9373.613 Da, which was nearly identical to the theoretical mass (i.e., 9372.86 Da) (Figure 1a). ^1^H-NMR spectra showed peaks mainly at three regions: 2.1–1.1, 4.2–2.7, and 8.6–7.8 ppm. Three peaks between 2.1 and 1.1 ppm can be attributed to methylene protons of lysine marked “b”, “c”, and “d” (Figure 1b). The chemical environment provided by the nearby amino group shifts methylene proton “‘e” signal towards 2.7–2.9 ppm. The peak at 4.2 ppm represents the proton in the CH group attached to an amine, marked as “a”. The peak at 13.5 ppm may be attributed to the proton of NH_2_ marked “h”. The peaks in the region 8.9–7.5 ppm represent the amino protons of the lysine sidechain, which is “g” in the figure.

Fourier transform infrared (FTIR) spectra of P_4_LDN (Figure 1c) also showed a profile of a typical peptide dendrimer structure. Peaks in the region 3500–3400 cm^−1^ corresponded to the –NH stretch of primary amines, and the peak at approximately 3400–3300 cm^−1^ corresponded to the –NH stretch of secondary amines. The peak in the range 3000–2840 cm^−1^ represented the C–H stretch of alkanes. The peak corresponding to 1690–1640 cm^−1^ indicated the C=O stretch of CO–NH (amide I band), and the peak corresponding to 1640–1550 cm^−1^ represented the N–H bends of CO–NH (amide II band) of the peptide.

The transmission electron microscopic (TEM) analysis at 80 kV showed that the P_4_LDN particles were spherical, and their size was approximately 64 ± 4.1 nm (Figure 1d).

### 3.2. Selection of the Best Short Peptide to Target EGFR In Silico and In Vitro

The structure of the EGFR–EGF complex was obtained from the Protein Data Bank (PDB id: 1NQL). The receptor–ligand binding region was identified, and the EGF residues that took part in the binding were identified as CNCVVGYIGER. Protein structure analysis and generation of structural images were performed using PyMOL. The average contribution of these EGF residues to the binding energy was then calculated after MD simulation (Table S1). It was observed that the amino acids, C-31, C-33, G-36, and G-39, showed much contribution to the binding energy. The amino acids that showed less contribution to the binding energy were replaced by other amino acids having similar characteristics. It was observed that the replacement of cysteines C-31 and C-33 with the amino acids alanine (A) and serine (S), respectively, resulted in higher binding (in the case of peptide E2). Then, the preliminary docking studies were conducted using PatchDock software. During the docking procedure, both the receptor and the peptide were considered as rigid, and the result was represented with the most favorable free energy of binding. The pose with lowest binding energy (or high binding affinity) was extracted and aligned with receptor structure for further analysis with Rosetta FlexPepDock. Table S2 shows the binding energies of the best five peptides after docking (Rosetta FlexPepDock) with the root mean square deviations (RMSD) and binding energies of these five peptides after MD simulation using GROMACS 5.1.4 software. All the five peptides bound to the similar binding site of EGFR like the natural ligand EGF (Figure S1).

These best five peptides were then synthesized manually using solid-phase peptide synthesis by fmoc strategy, purified, and characterized by HPLC and MALDI-TOF MS (Figure S2). Their binding efficacy studies were performed in vitro (Figures S3–S7), and we observed that out of the five peptides, peptide E2 showed the highest binding efficacy.

### 3.3. Characterization of MTX–G–P_4_LDN–E2 

**Scheme 2:**
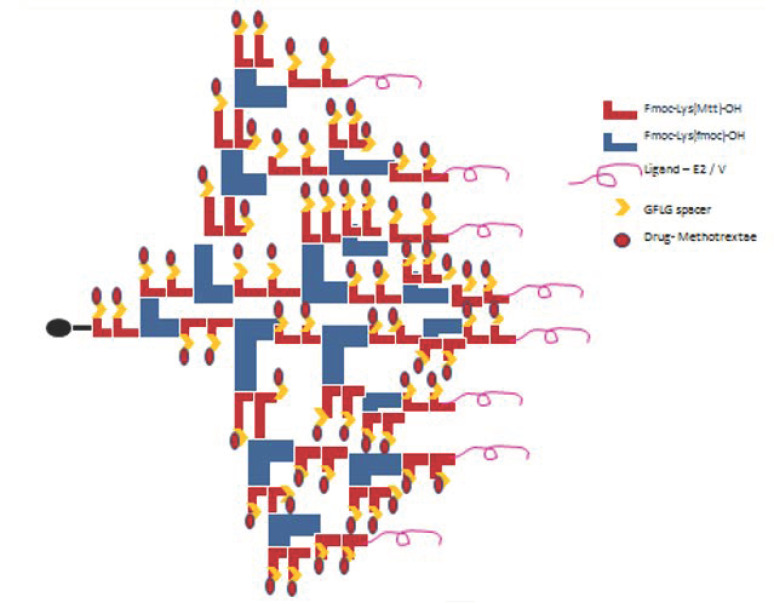
Attachment of EGFR-targeting peptide ligand (E2), GFLG spacer (G), and the drug MTX to P_4_LDN.

**Scheme 3:**
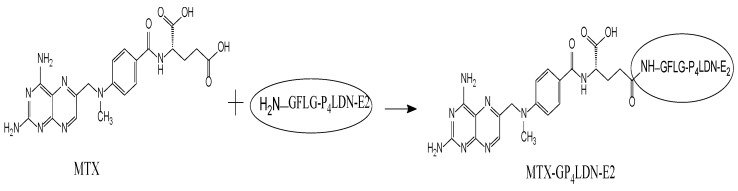
Attachment of MTX to G–P_4_LDN–E2.

**Figure 2:**
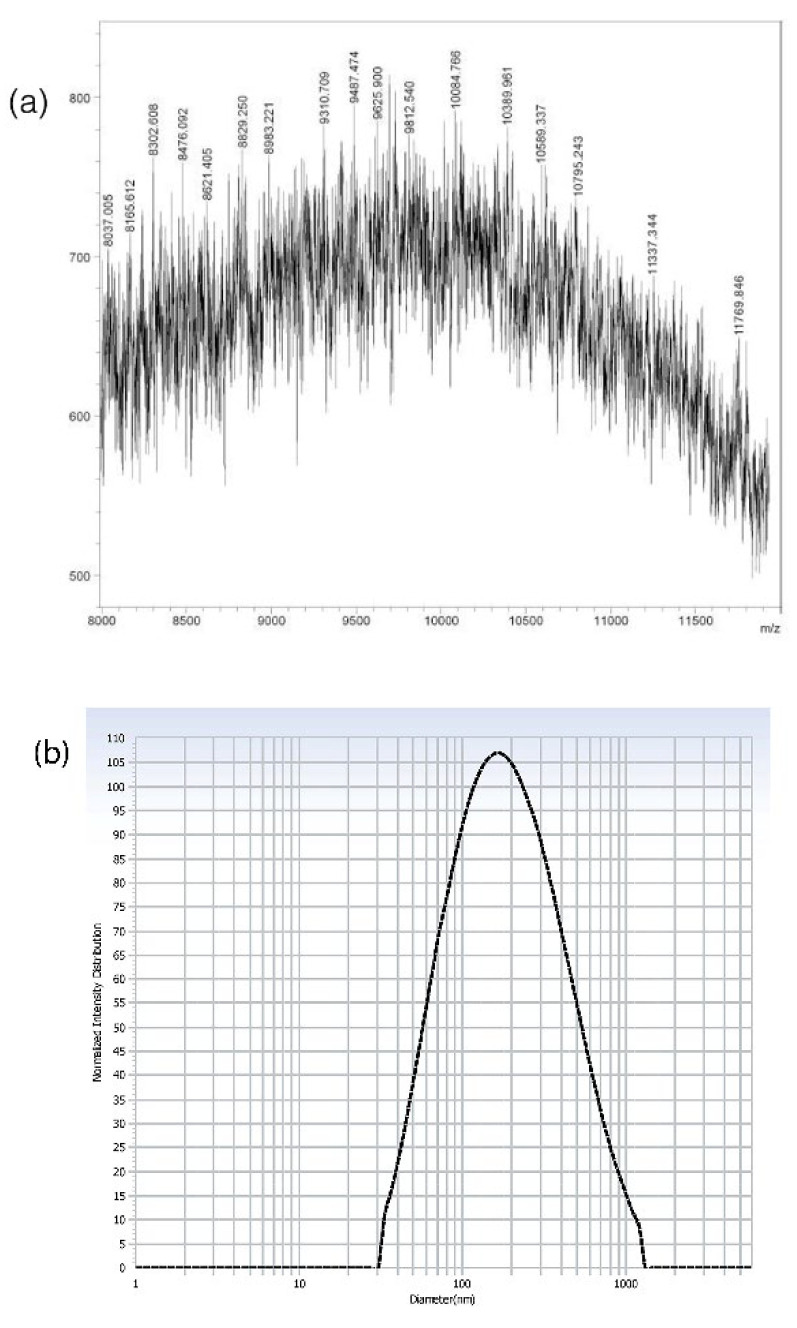

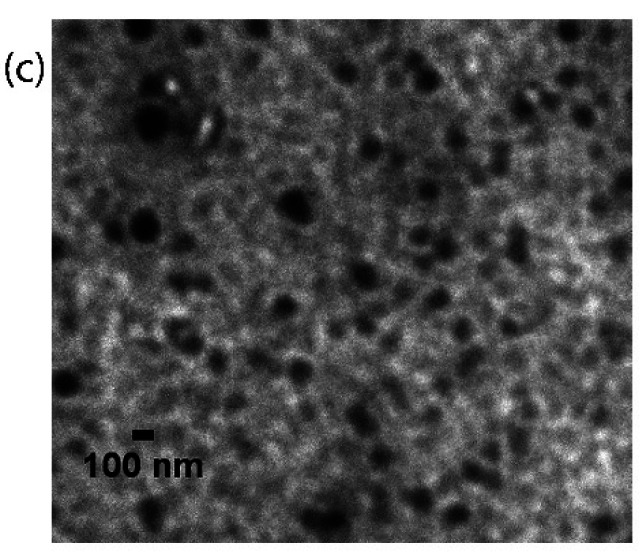
(**a**) MALDI-TOF MS profile: The [M+H]^+^ value calculated for C547H1147N163O83 was 11,336.96 Da and found to be 11,337.344 Da; (**b**) hydrodynamic size distribution pattern; (**c**) TEM image of MTX–G–P_4_LDN–E2 (scale bar: 100 nm).

After the synthesis of P_4_LDN, the peptide E2 was incorporated to half of the terminal amino groups of P_4_LDN. The anticancer drug, MTX, was then incorporated into the rest of the amino functionality of P_4_LDN through a tetrapeptide linker (G) (Scheme 2 and Scheme 3). The drug delivery system, MTX–G–P_4_LDN–E2, was cleaved from the resin, precipitated using ice-cold DEE, centrifuged, and lyophilized. They were then purified by C18 RP-HPLC and characterized by MALDI-TOF MS (Figure 2a).

From MALDI spectra, the observed mass of MTX–G–P_4_LDN–E2 was 11,337.344 Da, which was similar to their theoretical mass (i.e., 11,336.96 Da). The hydrodynamic particle size and polydispersity index of MTX–G–P_4_LDN–E2 were found to be 230.4 ± 12 nm and 0.268 ± 0.13, respectively. The zeta potential of the DDS was observed as +1.56 ± 0.2. The morphologies of the particles were analyzed by TEM and the size of MTX–G–P_4_LDN–E2 was observed to be approximately 134 ± 5.8 nm, and the particles were spherical in shape (Figure 2c).

### 3.4. Determination of Drug Concentration in the Nanocarrier System

The amount of loaded drug in the dendritic nanocarrier system was quantified by C18 RP-HPLC, and the peak area was determined. Then, the concentration of the drug was calculated using the standard MTX graph. The concentration of MTX in MTX–G–P_4_LDN–E2 was found to be 58 ± 3.4 mg/100 mg. 

### 3.5. Enzymatic Drug Release Assay

**Figure 3:**
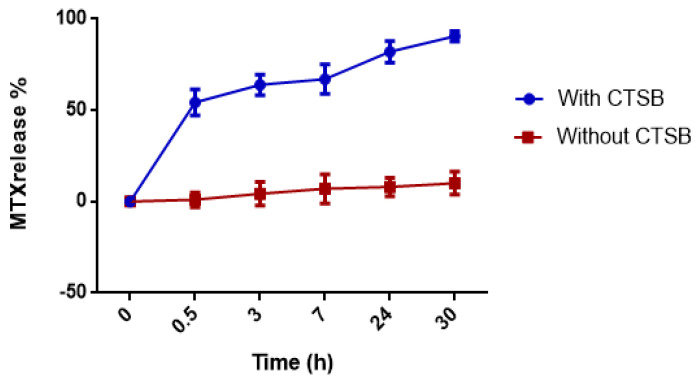
Time-dependent release of MTX from MTX–G–P_4_LDN–E2. The experiment was conducted in duplicate, and the data are represented as the arithmetic mean ± SD.

The drug release studies from the nanocarrier system with and without CTSB were carried out. It was observed that only less than 10% of the drug was released from the system in the absence of the enzyme at 30 h (Figure 3). 

In the presence of the enzyme, approximately 55% of the drug was released in 0.5 h, and the drug was almost completely (90%) released in 30 h.

### 3.6. Evaluation of the Receptor Binding Abilities of MTX–G–P_4_LDN–E2

The receptor binding abilities of MTX–G–P_4_LDN–E2 was evaluated by flow cytometry. Fluorescein MTX (5 μg/mL) and MTX–G–P_4_LDN–E2 (8.6 μg/mL) (having equivalent drug concentrations) were incubated with the receptor positive and negative cells for 24 h.

**Figure 4:**
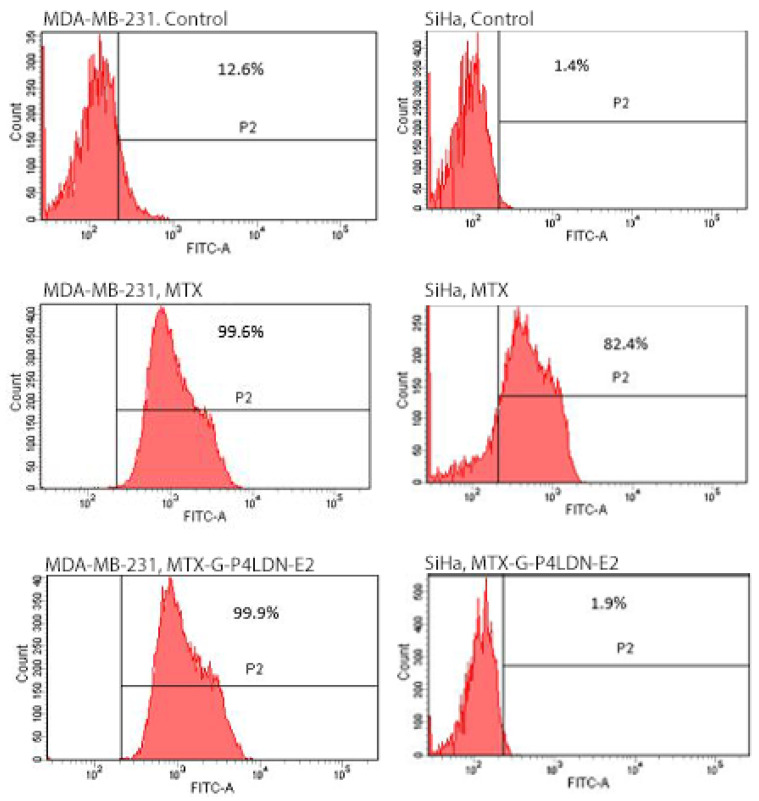
Binding abilities of MTX and MTX–G–P_4_LDN–E2 after 24 h of incubation with MDA-MB-231 (EGFR overexpressed) and SiHa (EGFR negative) cells. The cells without any treatment with MTX or the MTX-conjugated dendritic nanocarrier system were taken as the control.

Flow cytometry data (Figure 4) show that both the free drug as well as the nanocarrier system displayed similar binding abilities (>99%) in MDA-MB-231 cells. In the receptor negative SiHa cell, the free drug bound with 82.4% of cells, whereas the nanocarrier system bound with only 1.9% of cells.

### 3.7. Functional Evaluation of MTX–G–P_4_LDN–E2 

**Figure 5:**
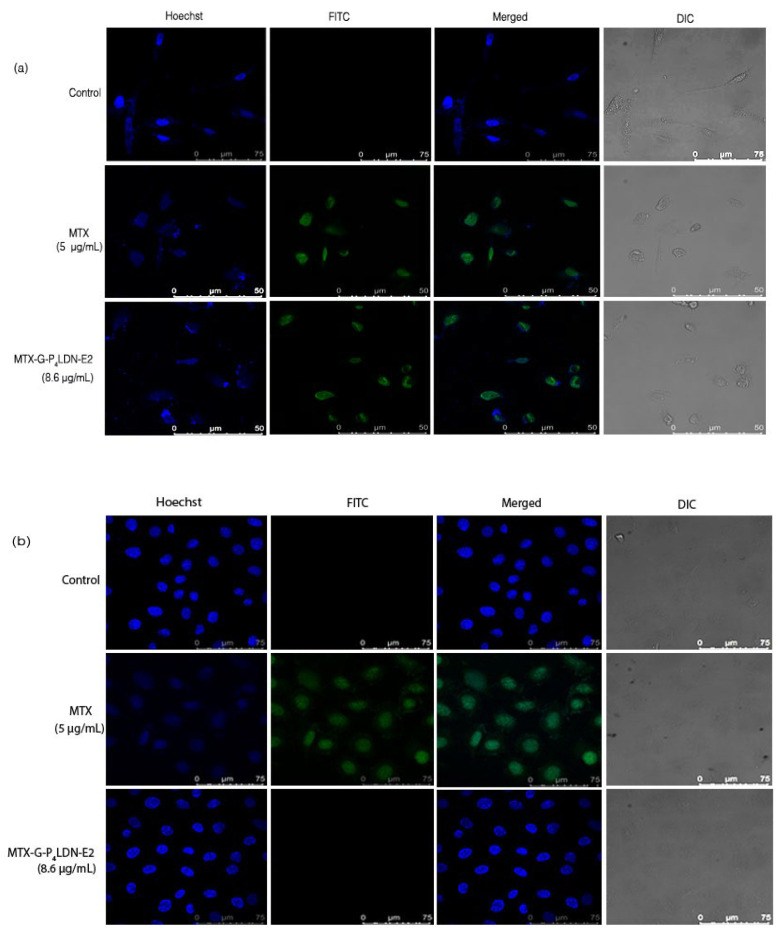
Confocal microscopy images of (**a**) MDA-MB-231 (EGFR overexpressed) and (**b**) SiHa (EGFR negative) cells after 24 h incubation with MTX and MTX–G–P_4_LDN–E2.

The binding ability and the cytotoxic nature of the system was evaluated using confocal microscopy. The receptor overexpressed as well as negative cells were incubated with fluorescein MTX (5 μg/mL) and fluorescein MTX-conjugated nanocarrier system (8.6 μg/mL) (having equivalent drug concentrations) for 24 h. As is obvious from the images (Figure 5), the free drug as well as the nanocarrier system bound with the MDA-MB-231 cells similarly, but in SiHa cells, only the free drug showed fluorescence. Moreover, the MDA-MB-231 cells treated with MTX as well as MTX–G–P_4_LDN–E2 had less fluorescence in Hoechst staining compared to control cells, which indicated the nuclei was damaged, whereas in case of SiHa cells, only the cells treated with MTX showed less fluorescence in Hoechst staining and, hence, damaged nuclei.

### 3.8. Internalization of MTX–G–P_4_LDN–E2

**Figure 6:**
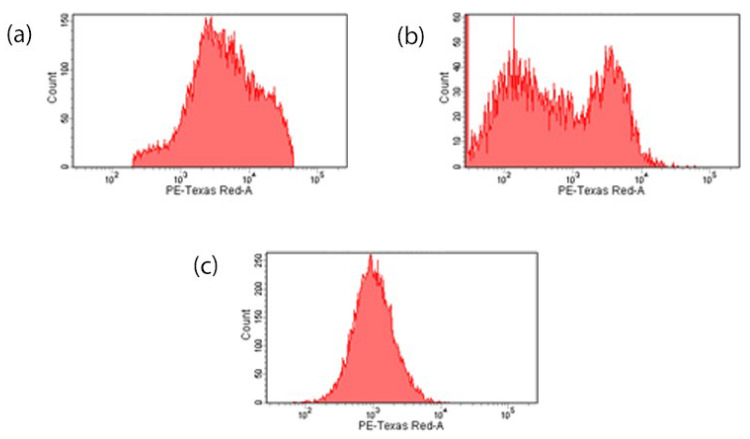

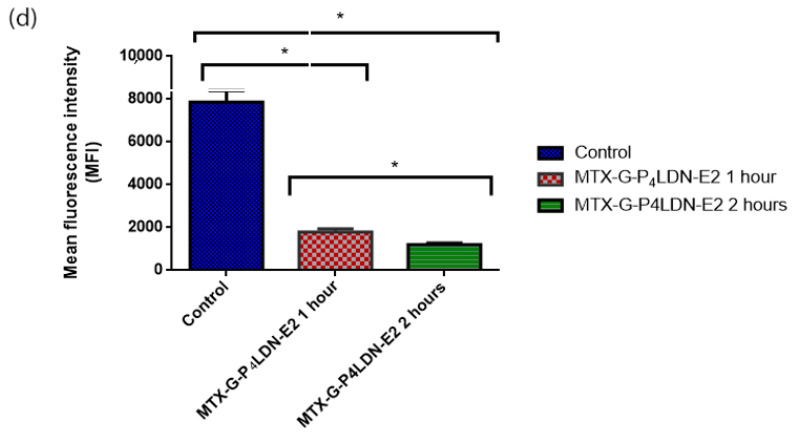
Internalization assay: (**a**) control cells without a nanocarrier system; (**b**) 1 h incubation of MTX–G–P_4_LDN–E2; (**c**) 2 h incubation of MTX–G–P_4_LDN–E2 with cells; (**d**) mean fluorescence intensity of cells. The experiments were conducted in duplicate, and the data are represented as the arithmetic mean ± SD. The statistical significance was measured using one-way ANOVA followed by Tukey’s test (* *p* < 0.005).

The preliminary evaluation of internalization was conducted by flow cytometry to analyze whether the nanocarrier system was internalized into the cell through the EGFR. MDA-MB-231 cells were incubated with fluorescein–MTX incorporated MTX–G–P_4_LDN–E2 (50 µg/mL) for 1 and 2 h. Figure 6 demonstrated that the nanocarrier bound to EGFR and internalized after incubating the cells with the nanocarrier system. The mean fluorescence intensity (MFI) of the control cells was 7861 ± 543.058, and after 1 h of incubation with MTX–G–P_4_LDN–E2, it reduced to 1792.5 ± 149.1995, and after 2 h of incubation, it further reduced to 1200 ± 77.78175.

### 3.9. Cytotoxicity Analysis of MTX–G–P_4_LDN–E2 

**Figure 7:**
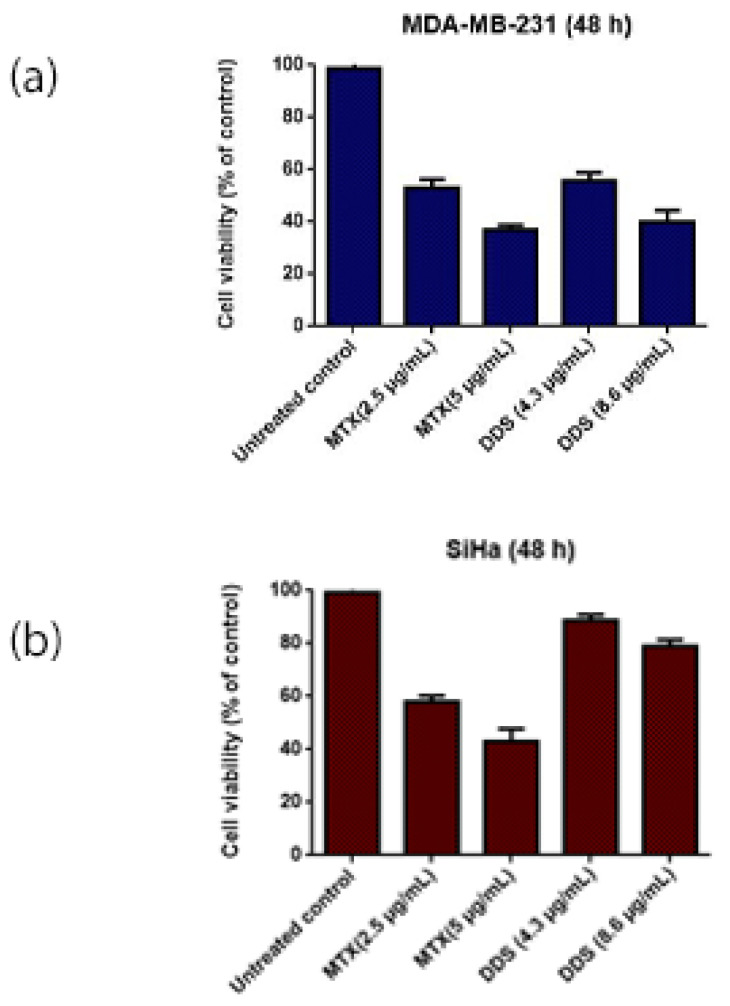
Cytotoxicity analysis of DDS by MTT assay: (**a**) MDA-MB-231 cells and (**b**) SiHa cells. The experiment was conducted in triplicate, and the data are represented as the arithmetic mean ± SD.

The cytotoxic nature of MTX–G–P_4_LDN–E2 at two different concentrations was analyzed using an MTT assay (Figure 7). It was observed that in the receptor positive cells, the nanocarrier system showed a similar cytotoxicity as the free drug at both concentrations used. The drug alone (at 5 µg/mL) resulted in 63% cytotoxicity, and the nanocarrier system at an equivalent drug concentration showed 59.95% cytotoxicity. But in the case of receptor negative cells, the free drug (at 5 µg/mL) resulted in 57% cytotoxicity, whereas the nanocarrier system showed only 20.95% cytotoxicity at an equivalent drug concentration. 

### 3.10. Analysis of the Cell Cycle Pattern

**Figure 8:**
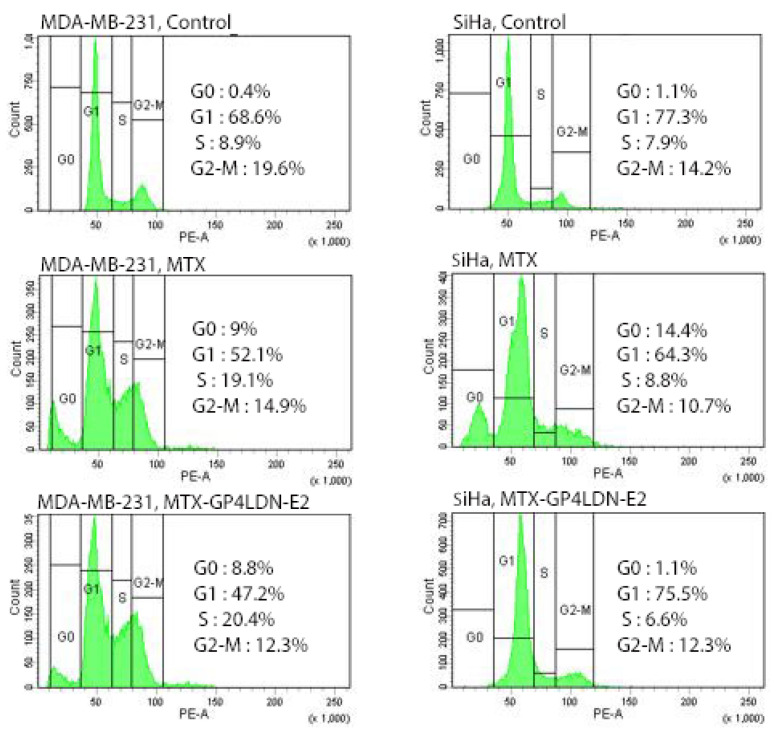
Cell cycle and cell death analysis by FACS in MDA-MB-231(EGFR overexpressed) and SiHa (EGFR negative) cells after 24 h incubation with MTX and MTX–G–P_4_LDN–E2.

Figure 8 shows the effect of MTX and the nanocarrier system on the cell cycle progression of MDA-MB-231 cells and SiHa cells. Compared to the control, MTX decreased the percentage of cells in the G2/M phase and increased the percentage of cells in the S and G0 phase in both these cells. In MDA-MB-231 cells, MTX–G–P_4_LDN–E2 produced a similar cell cycle pattern as that of MTX. But the nanocarrier system did not result in G2/M reduction as well as an S phase halt in the receptor negative cells. Moreover, the G0 fraction in receptor negative SiHa cells also did not increase.

### 3.11. Anti-Tumor Efficacy of MTX–G–P_4_LDN–E2 In Vivo

The treatment was started when the tumor volume reached 500–1000 mm^3^. There were 3 groups (*n* = 3 for control group and *n* = 6 for treatment groups). First group received PBS (5 mL/kg body weight) was treated as the control. Second and third group was given MTX (100 μL; 20 mg/kg) and MTX–G–P_4_LDN–E2 (100 μL; 35 mg/kg; equivalent MTX concentration), respectively. The treatment was given five times for 20 days. On every injection day, the tumor volume as well as the body weight of the animals were measured.

**Figure 9:**
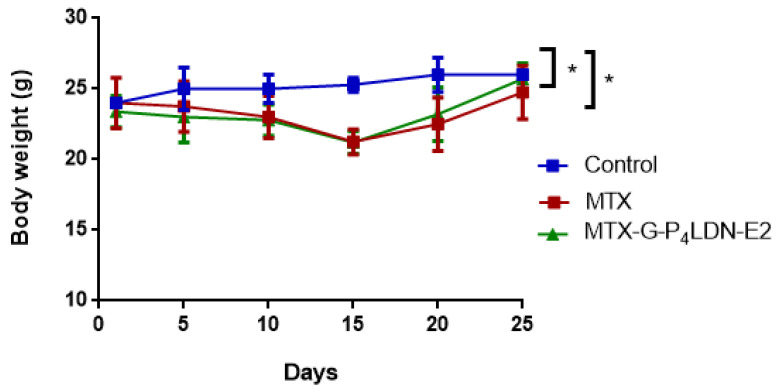
Effect of tumor treatment on body weight in female NOD-SCID mice (*n* = 3 for control group, *n* = 6 for MTX, and *n* = 6 for MTX–G–P_4_LDN–E2). The data are represented as the arithmetic mean ± SD. Statistical analysis was performed using one-way ANOVA followed by Dunnett’s test (* *p* < 0.05 between control and treatment groups).

**Figure 10:**
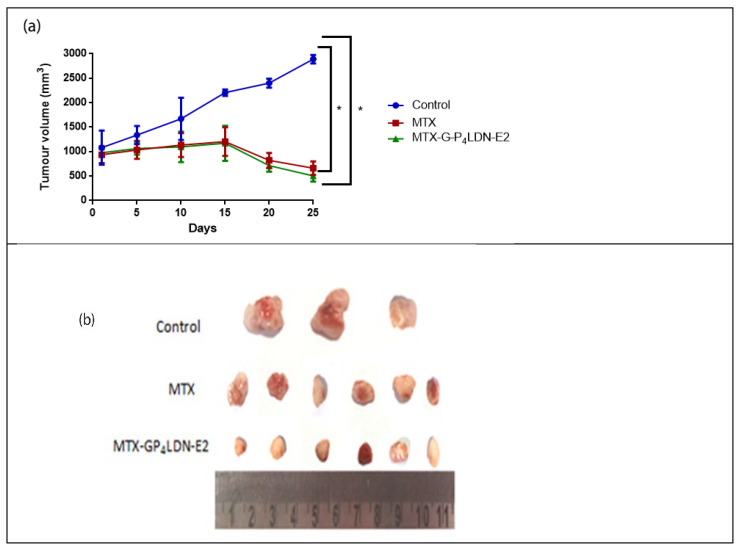
(**a**) Anti-tumor efficacy of MTX (20 mg/kg) and MTX–G–P_4_LDN–E2 (35 mg/kg) in female NOD-SCID mice (*n* = 3 for the control group, *n* = 6 for MTX, and *n* = 6 for MTX–G–P_4_LDN–E2). Data are represented as the arithmetic mean ± SD, and one-way ANOVA followed by Dunnett’s test were performed for statistical analysis (* *p* < 0.005). (**b**) Comparison of tumor volumes between the control and treatment groups.

Figure 9 shows the body weight pattern of mice. It was observed that in the control group, a gradual increase in body weight occurred as the tumor volume increased. The animals in the treatment groups showed a reduction in the body weight up to day 10, and they regained their body weight after that. Moreover, the animals that received the nanocarrier DDS regained weight more rapidly than MTX. Figure 10 shows the effect of the drug and the nanocarrier system in the tumor treatment. Compared to the control group, both the treatment groups showed a significant reduction in tumor volume when measured on day 25. The average tumor volume in control group at the end of the treatment was 2890 ± 85.440 mm^3^, whereas it was 661.8 ± 209.067 mm^3^ (77.2% reduction) in the MTX-treated group and 503.4 ± 273.743 mm^3^ (82.6% reduction) in MTX–G–P_4_LDN–E2 treated group.

## 4. Discussion

The advantage of targeted drug delivery in cancer using nanocarriers/nanoparticles over conventional chemotherapy includes minimum toxicity and maximum efficacy. This could be the result of controlled drug release and improved pharmacokinetics and pharmacodynamics. A suitable drug carrier is important to ensure site-specific sustained drug delivery. The invention of the most suitable DDS for cancer treatment with minimum limitations is inevitable to trim down the substantial increase in cancer cases. In this study, we developed a fourth-generation poly-lysine dendritic nanocarrier onto which targeting moieties were covalently incorporated to half of the N-terminal amino groups and the drug MTX to the remaining N-terminal amino groups and sidechain amino groups of all the lysine units with the help of a GFLG spacer. The targeting moiety used here was a short peptide that can target EGFR overexpressed breast cancers. As the whole EGF peptide consists of 53 amino acids, we designed short peptides that have more binding ability to bind with the receptor. Flow cytometry data and confocal images proved to have the highest binding nature of the peptide E2 with EGFR, and these peptides were highly receptor specific, as they did not bind with receptor negative cells (Supplementary Materials file). The drug release studies of MTX–G–P_4_LDN–E2 demonstrated the gradual release of the drug in the presence of the enzyme cathepsin B. This enzyme belongs to a family of papain-like proteases, and it is usually found in the lysosome, whereas it is upregulated in cancers [22,23]. In cancers, the enzyme is found in lysosomes as well as in vesicles throughout the cytoplasm and also at the cell periphery [24]. Cancer cells exhibit a slightly acidic environment that is very much suitable for the optimal activity of this enzyme [25]. The function of the enzyme is also well conserved among various species facilitating in vivo preclinical studies of GFLG spacer-based drug delivery. The sudden release of the drug in 0.5 h and a gradual increase afterwards may be due to the abundance of the enzyme in the initial period and a reduction in the concentration later. Drug release studies confirmed the unique enzyme sensitivity of the DDS. The size, morphology, and surface charge of the drug delivery system were also analyzed. The hydrodynamic size was larger than the size obtained from the TEM analysis, because DLS provides the size of the nanoparticle plus the liquid layer surrounding it, whereas TEM provides the particle size of the dried sample. The surface charge was found to be slightly positive despite the highly positive nature of lysine dendrimers. This reduction in the positive charge was due to the attachment of drug and targeting moieties to the terminal amino groups, which masked the bulky positive charge of lysine amino terminals. The efficacy of our DDS was analyzed by flow cytometry and confocal imaging and the cytotoxic ability was evaluated using MTT assay and cell cycle analysis. Confocal images (Figure 5) showed that the drug exerted its action in the receptor overexpressed cells in the free state and also when conjugated with the carrier. Hence, the nuclei were not intact, and the Hoechst staining showed reduced blue fluorescence in both the cases compared to the control. But only the free drug (i.e., MTX) acted with the receptor negative cells and resulted in cell damage, whereas the nanocarrier system did not bind with the receptor negative cells (cells were also intact). This further confirmed that the MTX is non-specific in action, whereas the nanocarrier system is specific to the receptor overexpressed cells (consistent with the flow cytometry results). All these experiments confirmed the targeted action of our DDS in the cancer cells that overexpress the receptors. The preliminary studies regarding the internalization of the nanocarrier system suggest that the DDS was internalized through EGFR. Cytotoxicity evaluation by MTT dye clearly indicated the non-specific cytotoxic action of the drug MTX, whereas the nanocarrier system was specific to EGFR, and the drug from the system was released only in receptor overexpressed cells. This further confirmed that the absence of receptor seriously affected the targeting ability, thereby reducing the cytotoxicity of the nanocarrier system. 

MTX is an antimetabolite that interferes with folate metabolism and DNA synthesis. It is a folate analog that competitively inhibits dihydrofolate reductase (DHFR), an enzyme that participates in the tetrahydrofolate synthesis [26]. It induces cell cycle arrest at S phase and results in the reduction of cells in the G2/M phase. The cell death and cell cycle analysis by flow cytometry indicated that the DDS did not exert its action in receptor negative cells. This clearly pointed out that the peptide ligand and the spacer provided the required target specificity to the DDS. The efficacy analysis of the DDS was also studied in NOD-SCID mice after developing tumors. Compared to the control, there was a substantial reduction in tumor volume in the case of DDS than free drug, which further confirmed the therapeutic efficacy of the system. The mice that received the DDS appeared to gain weight more rapidly, suggesting that the dendrimer was well tolerated by mice compared to free MTX. All of the analyses showed the superior efficacy of the nanocarrier system MTX–G–P_4_LDN–E2 over the free drug. Although we performed some in vitro and in vivo studies to prove the efficacy of the poly-lysine dendritic nanocarrier system to deliver MTX, only a preliminary study regarding the internalization of the system was carried out. The mode of internalization (i.e., clathrin- or caveolin-dependent or independent), concentration dependent internalization, and prolonged time internalization studies must be explored in the future. Immunogenicity studies should also be executed before moving to clinical trials. A small limitation of this study was the selection of the receptor (EGFR) because they are not specific to cancer, and they are also present in normal cells in minor quantities; hence, our system will also approach normal cells. Thus, identification of cancer cell-specific receptors and the design and incorporation of peptide ligands that target such receptors should be executed in the future so that the specificity can be further increased. However, this study sheds light on the use of peptide-conjugated poly-lysine dendrimers for cancer drug delivery, which can be a breakthrough in the nano era.

## Data Availability

All the data presented in this study are provided within the main text and supplementary information.

## Supplementary Materials

The following are available online at https://www.mdpi.com/article/10.3390/ma15030800/s1. Table S1. MD simulation results; Table S2. Binding energies of the peptides from Rosetta FlexPepDock with RMS deviation and binding energies after MD simulation using GROMACS 5.1.4 software; Figure S1. Docking results after MD simulation with GROMACS 5.1.4; Figure S2. MALDI-TOF-MS peaks of Rhodamine-B dye-conjugated EGFR--targeting short peptides; Figure S3. Expression of EGFR in MDA-MB-231 cells; Figure S4. Binding activities of rho B-conjugated peptides after incubating with MDA-MB-231 cells for 2 h at 37 °C; Figure S5. Binding activities of rhodamine B-conjugated peptides after incubating with SiHa cells for 2 h at 37 °C; Figure S6. Internalization of peptides in MDA-MB-231 cells; Figure S7. Internalization of peptides in SiHa cells.
