# Supplementary material for: Poly-Lysine Dendritic Nanocarrier to Target Epidermal Growth Factor Receptor Overexpressed Breast Cancer for Methotrexate Delivery
Source: Materials (Basel). 2022 Jan 21;15(3):800. doi: 10.3390/ma15030800 (PMC8836561; doi:10.3390/ma15030800)
Supplement: Supplementary file 1 [file materials-15-00800-s001.zip › materials-1475699-supplementary.pdf]

materials

MDPI

Supplementary materials

Poly-Lysine Dendritic Nanocarrier to Target Epidermal Growth Factor Receptor Overexpressed Breast Cancer for Methotrexate Delivery

Pratibha Narayanan [1,2], Anju Krishnan Anitha [1,2], Neethu Ajayakumar [1,2] and Kesavakurup Santhosh Kumar [1,*]

[1] Pathogen Biology Division, Rajiv Gandhi Centre for Biotechnology, Jagathy, Poojappura P.O., Thiruvananthapuram 695014, Kerala, India; pratibhan@rgcb.res.in (P.N.); anjuka@rgcb.res.in (A.K.A.); neethua@rgcb.res.in (N.A.)

[2] University of Kerala, Thiruvananthapuram 695034, Kerala, India

* Correspondence: kskumar@rgcb.res.in; Tel.: +91471 2529518

Table S1. MD simulation results: The structure of EGFR-EGF complex was obtained from protein data bank (PDB id: 1NQL). The receptor-ligand binding region was identified and the EGF residues that take part in the binding was identified as CNCVVGYIGER. Average contribution of the EGF residues 31-41 (PDB id: 1NQL) in the binding energy with receptor EGFR was obtained as shown below.

Amino acid residue and its position in the ligand	Total energy ± standard deviation error
C-31	-36.5 ± 14.4
N-32	44.5 ± 11.1
C-33	-22.0 ± 10.6
V-34	85.9 ± 5.9
V-35	13.6 ± 7.3
G-36	-17.3 ± 8.0
Y-37	106.5 ± 11.7
I-38	8.3 ± 9.2
G-39	-62.9 ± 12.8
E-40	83.6 ± 11.2
R-41	51.1 ± 8.4

Table S2. Binding energies of the peptides from Rosetta FlexPepDock with RMS deviation and binding energies after MD simulation using Gromacs 5.1.4 software. The peptide E2 showed highest binding affinity (lowest energy) compared to other four novel short peptides.

Peptide name	Amino acid sequence	Free energy score from Rosetta FlexPepDock ab-initio (RMS BB)	Binding energy after MD simulation ($kJ.mol^{-1}$)
PDB id: 1NQL	CNCVVGYIGER	-82.688 (8.87)	-89.616 ± 67.393
E1	CVCHVGFTGAR	-94.347 (1.782)	-120.940 ± 46.103
E2	ARSHVGYTGAR	-93.668 (2.433)	-215.579 ± 73.531
E3	CVCHVGWTGAR	-88.138 (2.131)	-115.479 ± 63.809
E4	CVCHVGYTGVR	-90.981 (1.701)	- 107.980 ± 93.467
E5	CVCHVGWIGAR	-80.567 (8.887)	-102.993 ± 5.890

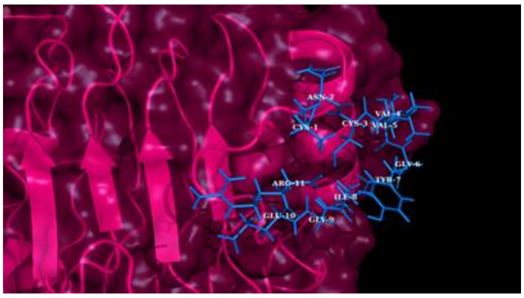

EGFR-1NQL

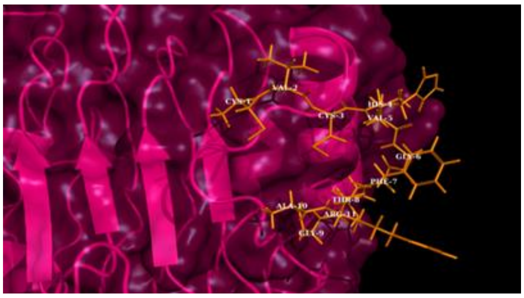

EGFR-E1

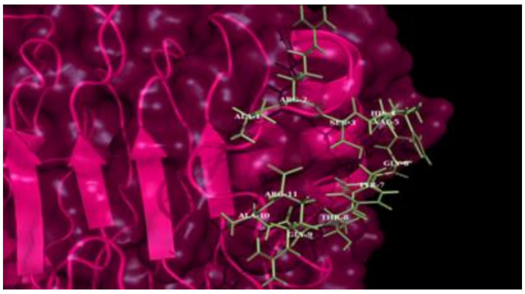

EGFR-E2

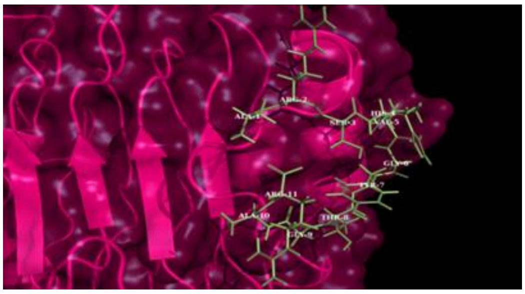

EGFR-E3

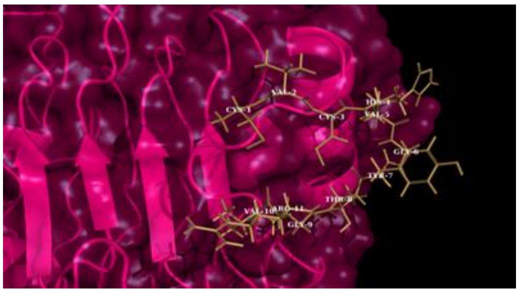

EGFR-E4

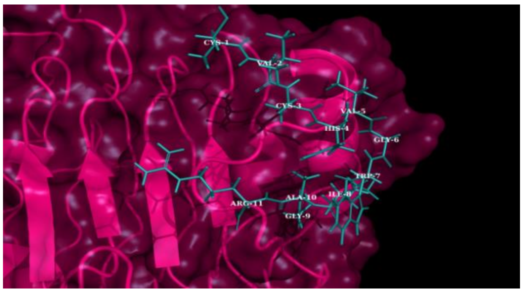

EGFR-E5

Figure S1. Docking results after MD-simulation with Gromacs 5.1.4 : Binding of peptide with EGFR with a time scale of 20ns. All the peptide ligands bind to the similar active site of EGFR as that of the natural EGF ligand (PDB id: 1NQL).

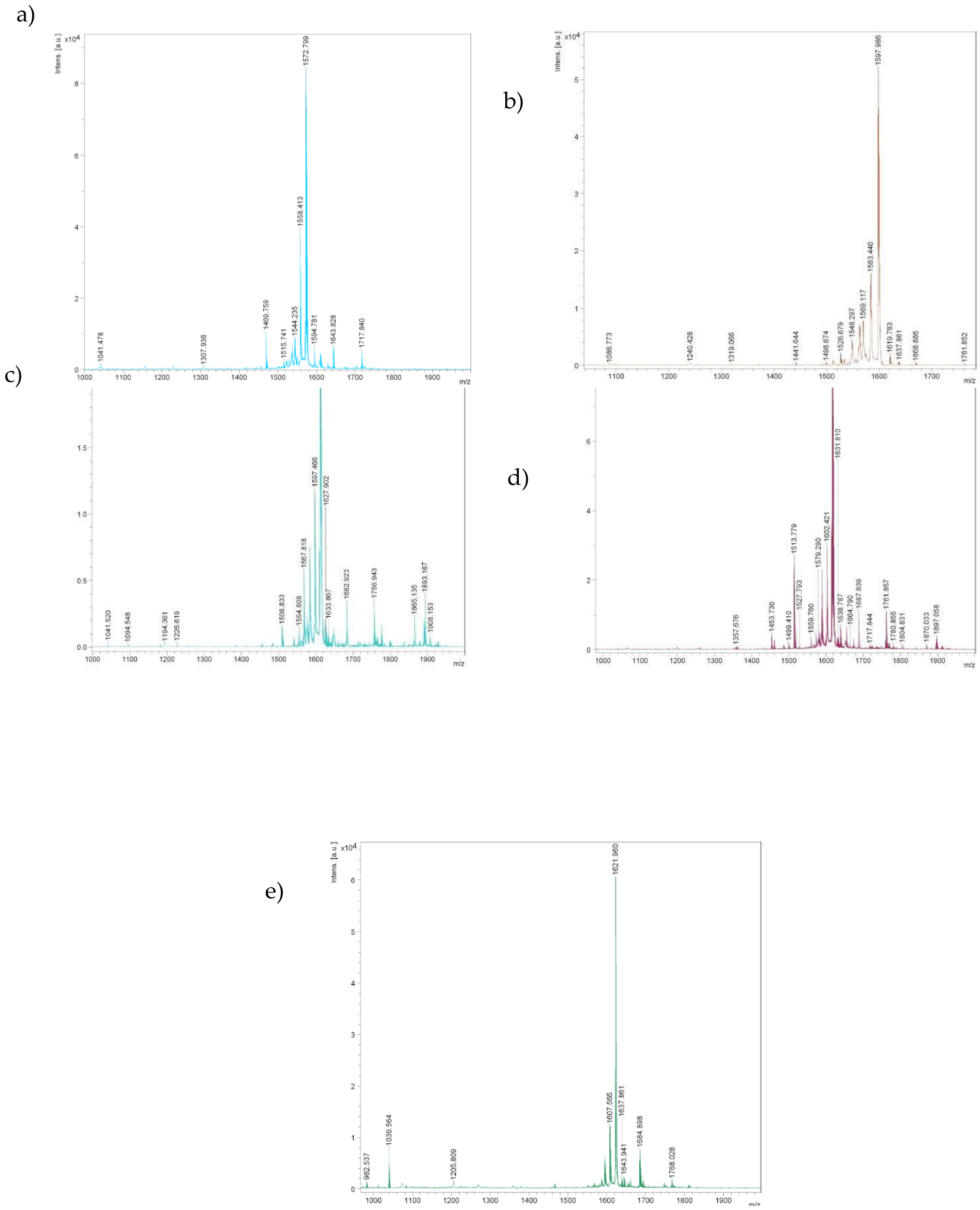

Figure S2. MALDI-TOF-MS peaks of Rhodamine-B dye conjugated EGFR targeting short peptides. a) E1-Rho B, b) E2-Rho B, c) E3-Rho B, d) E4- Rho B and e) E5-Rho B. The observed masses were similar to that of the theorectical masses.

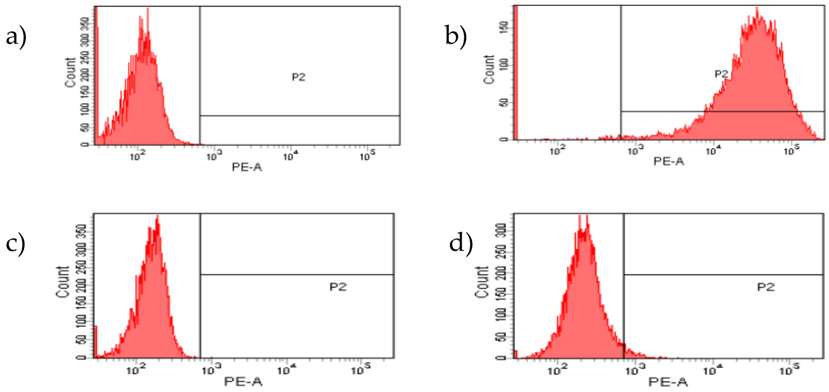

Figure S3. Expression of EGFR in MDA-MB-231 cells. a) untreated control and b) treated with PE conjugated antibody was confirmed by flow cytometry. Expression of EGFR on SiHa cell lines c) untreated control and d) treated with PE conjugated antibody was confirmed by flow cytometry.

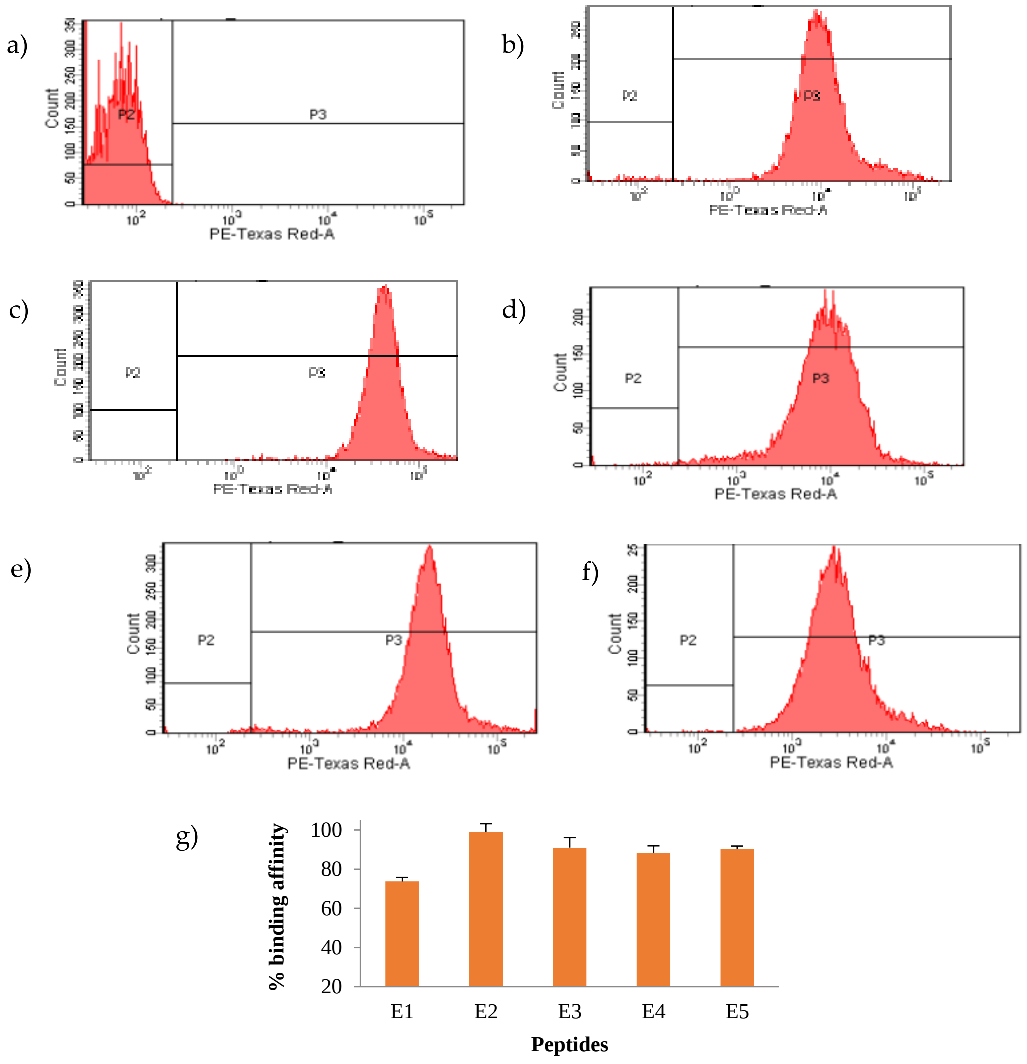

Figure S4. Binding activities of rho B-conjugated peptides after incubating with MDA-MB-231 cells for 2 hours at 37°C. a) Untreated control MDA-MB-231 cell. MDA-MB-231 cells binding with b) E1, c) E2, d) E3, e) E4, and f) E5. g) Binding affinity percentages of rho B- conjugated peptides.

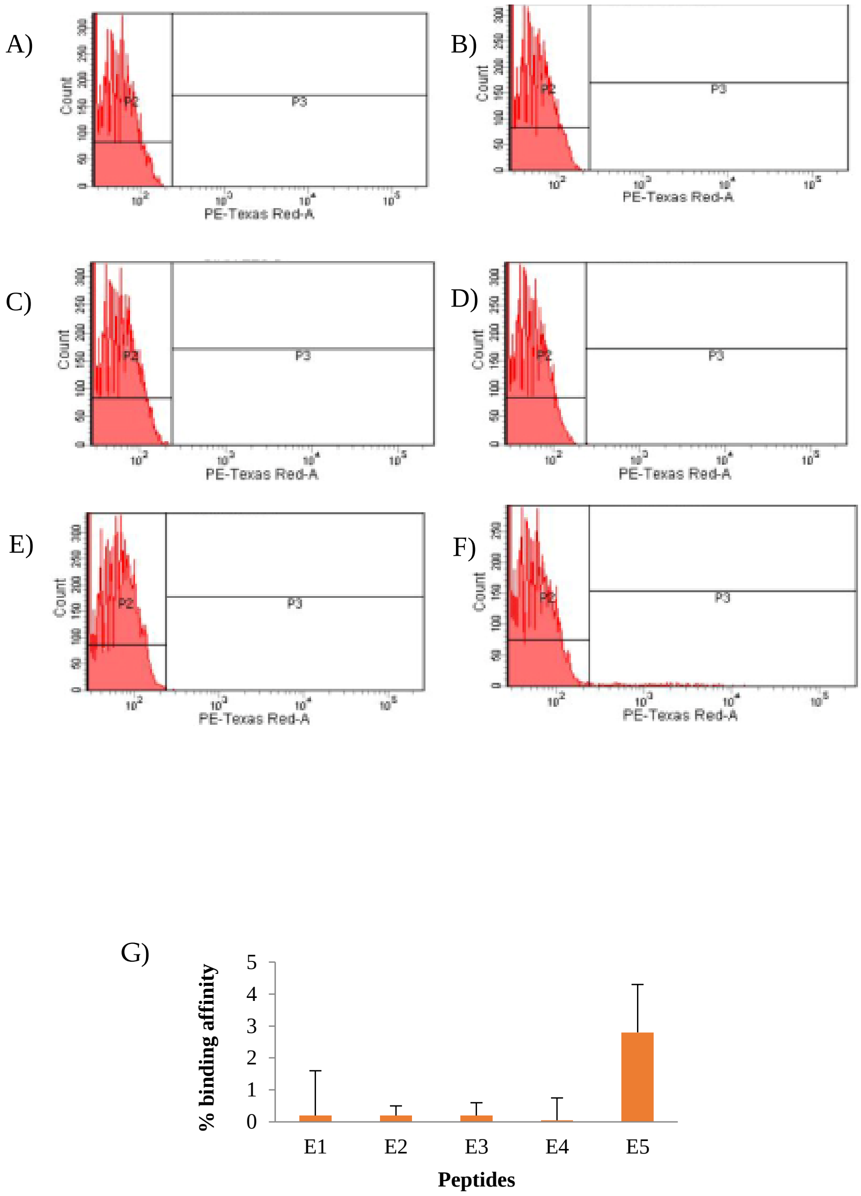

Figure S5. Binding activities of rhodamine B-conjugated peptides after incubating. **with SiHa cells for 2 hours at 37°C.** A) Untreated control SiHa cell. SiHa cells binding with B) E1, C) E2, D) E3, E) E4, and F) E5. G) Binding affinity percentages of rho B-conjugated peptides.

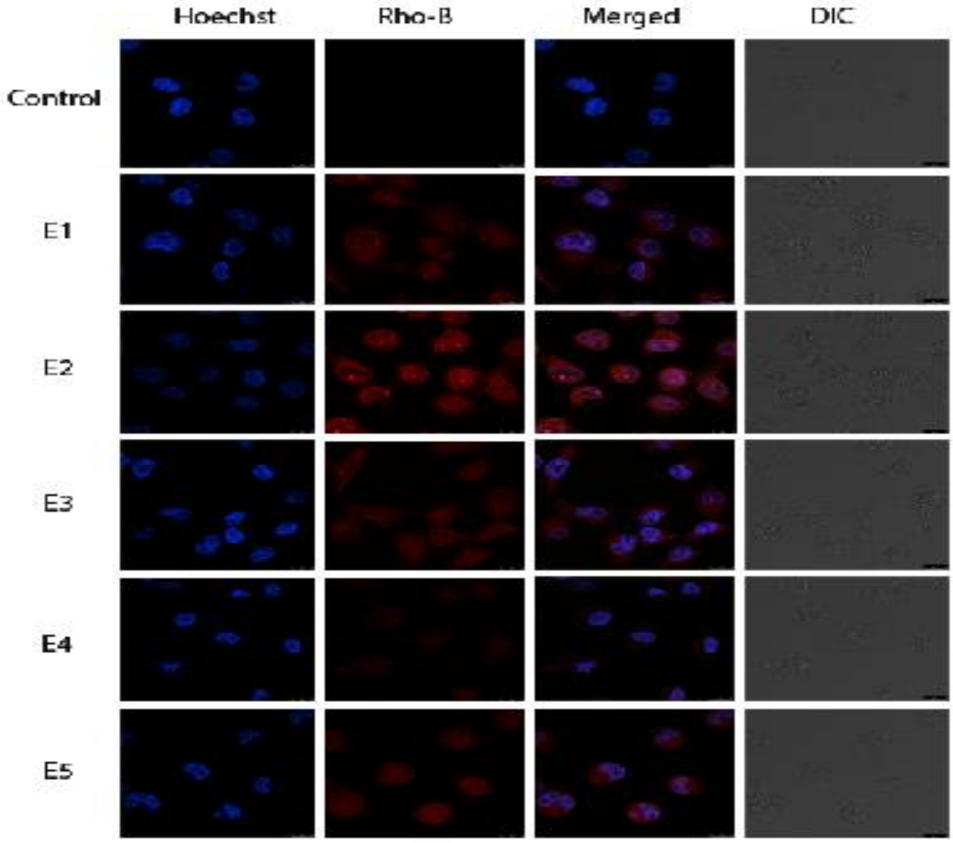

Figure S6. Internalization of peptides in MDA-MB-231 cells. MDA-MB-231 (EGFR overexpressed) cells were incubated with E1 to E5 peptides (50 μM) for 2 hours at 37°C. Rho-B alone was given as the control. The cells were nuclear stained with Hoechst stain.

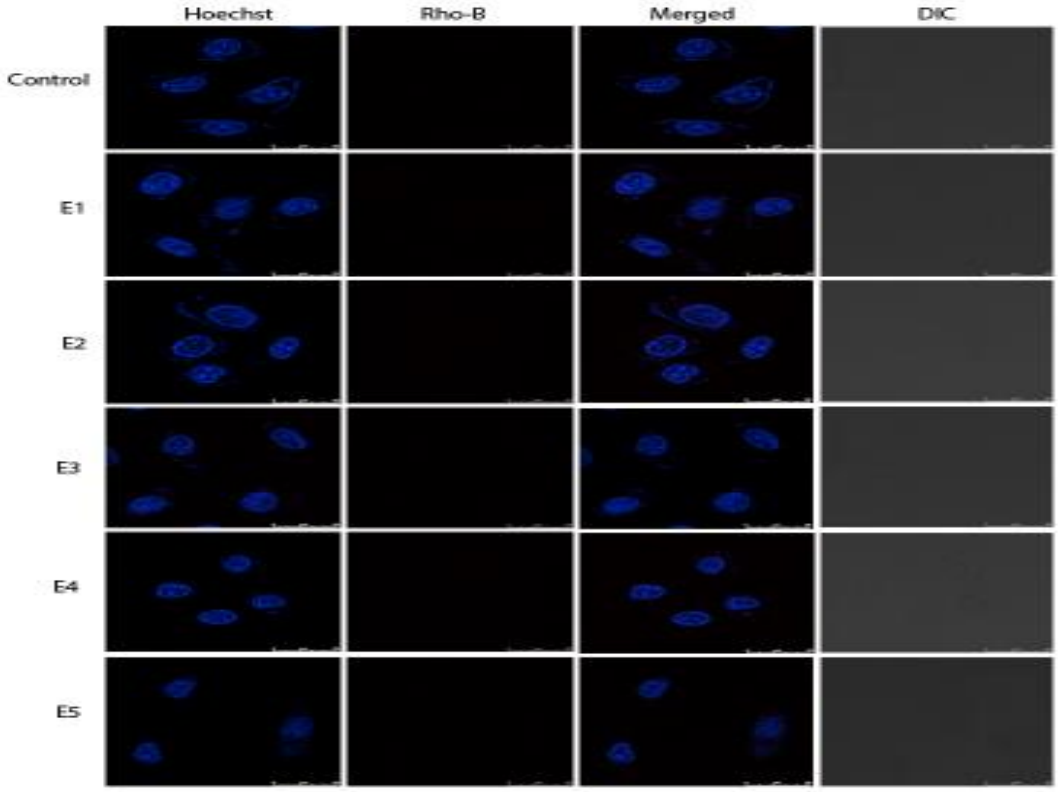

Figure S7. Internalization of peptides in SiHa cells. SiHa (EGFR negative) cells were incubated for 2 hours with E1 to E5 peptides (50 μM) for 2 hours at 37°C. Rho-B alone was given as the control. The cells were nuclear stained with Hoechst stain.
